# Dual-Band and Wideband Bandpass Filters Using Coupled Lines and Tri-Stepped Impedance Stubs

**DOI:** 10.3390/mi14061254

**Published:** 2023-06-14

**Authors:** Abdullah J. Alazemi

**Affiliations:** Electrical Engineering Department, College of Engineering and Petroleum, Kuwait University, Safat, Kuwait City 13060, Kuwait; aalazem.ku@ku.edu.kw

**Keywords:** coupled line, tri-stepped impedance stubs, bandpass filter, dual-band, wideband

## Abstract

In this paper, two bandpass filters—one with a dual-band response and the other with a wideband response—were designed, implemented, and experimented with. The filters are based on the novel combination of series coupled lines and tri-stepped impedance stubs. However, coupled lines along with tri-stepped impedance open stubs (TSIOSs) give a third-order dual passband response. The advantage of dual-band filters using coupled lines and TSIOSs is that they have wide passbands that are close together and separated by a single transmission zero. In contrast, the inclusion of tri-stepped impedance short-circuited stubs (TSISSs) instead of TSIOSs provides a fifth-order wide passband response. The advantage of wideband bandpass filters using coupled lines and TSISSs is that they have a very good selectivity factor. Theoretical analysis was carried out to validate both filter configurations. The tested bandpass filter using coupled lines and TSIOS units had two close wide passbands operating at 0.92 and 1.52 GHz center frequencies, respectively. The dual-band bandpass filter was implemented to operate in GSM and GPS applications. The first passband had a 3 dB fractional bandwidth (FBW) of 38.04%, while the second passband had a 3 dB FBW of 22.36%. The experimental result of the wideband bandpass filter (with coupled lines and TSISS units) had a center frequency of 1.51 GHz with a 3 dB fractional bandwidth of 62.91% and a selectivity factor of 0.90. A good congruence was demonstrated between the full-wave simulated and tested results for both filters.

## 1. Introduction

Bandpass filters are mainly transmission line-based passive devices that are used in many RF/microwave circuits and wireless communication and radar systems. They are essential front-end components that can pass signals within a certain frequency range and reject unwanted out-of-band signals [1,2,3,4,5,6,7,8,9,10,11,12,13,14,15,16,17,18,19,20,21,22,23,24,25,26,27,28,29,30,31,32,33,34]. There are many parameters to be considered in the design and implementation of a bandpass filter, such as the design structure, size, passband frequency bandwidth, insertion loss, selectivity, roll-off rate, isolation, and stopband rejection. One of the main obstacles in passive circuits in general, and in filters in particular, is the low-frequency bandwidth. Many technical solutions have been proposed to increase the filter’s frequency bandwidth, including the adoption of multiple frequency bands (dual, triple, quadruple, etc.) [1,2,3,4,5,6,7,8,9,10,11,12,13,14,15,16,17,18]. This type of filter is excellent for multiple frequency systems such as carrier aggregation or wireless local area network (WLAN) bands. Another method for obtaining a wideband response is by modifying the filter structure or attaching other elements to increase the bandwidth—such a method works well for wideband applications [19,20,21,22,23,24,25,26,27,28,29,30,31,32].

Many bandpass filters with different structures had been presented in the literature to achieve a dual-band response, such as employing a bandpass filter using split-ring resonators (SRRs) and stepped-impedance resonators (SIRs) [1]. Dual-band bandpass filters (DBBPFs) using modified hexagonal SSRs are demonstrated in [2], and triangular and rectangular loop resonators are shown in [3]. The DBBPFs based on these resonators occupy a smaller footprint; however, they have a narrow bandwidth. The stub-loaded theory has also been adopted for designing DBBPFs [4]. In [5], two resonators—a hairpin resonator and a meandering uniform impedance resonator—were loaded with stubs and utilized to develop a DBBPF with a narrow bandwidth. A symmetric open-circuited stub-loaded resonator was proposed in [6] to realize a DBBPF with a high isolation and wide stopband [6]. A DBBPF with a center frequency ratio of 1.15 was demonstrated using a stub-loaded resonator and two shorted lines coupled to a stub-loaded resonator in [7]. Apart from the above stub loading techniques, modified open/short-circuited stub-loaded resonators [8], quint-mode resonators [9], and ring resonators loaded with multi-stubs [10] have been employed to realize DBBPFs with good selectivity. Even though some of the DBBPFs based on the stub-loaded theory have a center frequency ratio of less than two, they have a narrow bandwidth. Compact DBBPF topologies can be obtained by using a modified SIR [11] or stepped impedance loaded by stubs [12]. Another approach for obtaining DBBPFs is by the use of coupled lines [13], or by using coupled microstrip rings [14] or parallel-coupled SIR-based dual-band bandpass filters [15]. Other techniques include broadside-coupled-based DBBPF [16] or signal interference/interaction techniques [17,18]. A shorted coupled line and a stepped impedance transmission line loaded with open stubs in an H-shaped configuration were utilized in [17] to demonstrate a paper substrate-based filter with a dual-band response. A DBBPF with wide passbands employing a simple transmission line in path 1 and a cascaded section of transmission lines and a shorted coupled line in path 2 is reported in [18]. DBBPFs based on SIRs, coupled lines, and signal interference techniques have high selectivity due to having a higher number of transmission zeros and their compact topology. However, their 3 dB fractional bandwidths are lower.

Single-band bandpass filters with a wideband response are another approach for increasing a filter’s bandwidth. Methods for obtaining a wideband response have been demonstrated in the literature and include the use of: folded multiple-mode resonators [19], dual-mode ring resonators [20], folded-arms square open-loop resonators [21], cross-shaped resonators [22], T-shaped resonators and L-shaped defected microstrip structures [23], and stepped impedance open stub-loaded ring resonators with a high selectivity response [24]. A bandpass filter with five poles in the passband and 58.3% 3 dB FBW was developed by employing a ring resonator loaded with stepped impedance open stubs [24]. A wideband bandpass topology consisting of shunt short-circuited stubs and half wavelength transmission lines was reported in [25]; the proposed filter configuration had a low selectivity factor of 0.62. The realized filter had five poles and 50% FBW. A wideband bandpass filter with combined short- and open-circuited stubs was proposed in [26]. Two pairs of transmission zeros (TZs) can be created on either side of the required passband using the two different types of stubs. Two transmission lines coupled over a length of 3 λ_g_/4 and quarter wavelength coupled line were employed in [27] to demonstrate a bandpass filter with eight transmission zeros and good out-of-band suppression. Several bandpass filters have been used with coupled lines to design single-band wideband passband filters [28,29,30,31]. In [28], two wideband bandpass filters employing cross-shaped resonators with open/short parallel coupled lines are reported. The 3 dB absolute bandwidth of both the implemented filters were 1.7 GHz and 0.7 GHz, respectively, and occupied the same footprint. In [29], a multi-stage-stepped impedance resonator (SIR) that generates multiple transmission zeros was capacitively coupled to another multi-stage SIR to realize an ultra-wideband bandpass filter with 97% 3 dB FBW. Coupled lines, shorted stubs, and transmission lines were utilized to realize a bandpass filter with 73.17% FBW; In addition, another bandpass filter with 78.34% FBW was realized using coupled lines and stepped impedance stubs in [30]. Coupled lines in a ring configuration loaded with open and short stubs was proposed in [31] for developing a bandpass filter. The proposed filter had very good out-of-band characteristics; however, the 3 dB FBW was only 23.7%. In [32,33], transversal signal-interaction concepts were employed to realize compact wideband bandpass filters with high selectivity. A fifth order bandpass filter utilizing coupled lines, shorted stubs, and stub-loaded coupled lines was reported in [32]. A wideband bandpass filter containing coupled lines in one path and stepped impedance stubs loaded with shorted stubs in another path was realized in [33]. A wideband bandpass unit with lumped LC resonators and distributed shunt microstrip open-ended stubs was reported in [34]. The developed bandpass unit was cascaded with a lowpass filter based on mixed, lumped, and distributed elements to achieve a wide stopband.

In this work, two different bandpass filters are proposed to obtain dual-band and wideband responses. The bandpass filter with a dual-band response is realized using series-coupled lines and a shunt tri-stepped impedance open stub, whereas the bandpass filter with a wideband response is constituted of series-coupled lines and a shunt tri-stepped impedance short-circuited stub. Even–odd mode analysis is employed, as both the filter circuits are symmetric, for obtaining the theoretical equations for the transmission zero frequencies. The highlights of the proposed work are as follows:

- The DBBPF based on coupled lines and TSIOSs had wide passbands with 3 dB FBWs of 38.04% and 22.36%.
- The center frequency ratio of the DBBPF was 1.65, indicating close passbands separated by a single transmission zero, with a rejection greater than 20 dB.
- The realized WBBPF using coupled lines and TSISSs had a very good selectivity factor of 0.90.

The article is structured as follows: in Section 2, the design and analysis of the proposed bandpass filters are demonstrated—this includes the detailed theoretical derivations of the filters’ S-parameters using an even and odd analysis. In Section 3, the filters’ prototypes are manufactured, and both simulated and measured results are presented. A comparison between the proposed filters and published works are also demonstrated based on different filter parameters. Lastly, the manuscript is concluded.

## 2. Design and Analysis of Proposed BPFs

(a) *DBBPF using coupled lines and TSIOSs*

**Figure 1:**
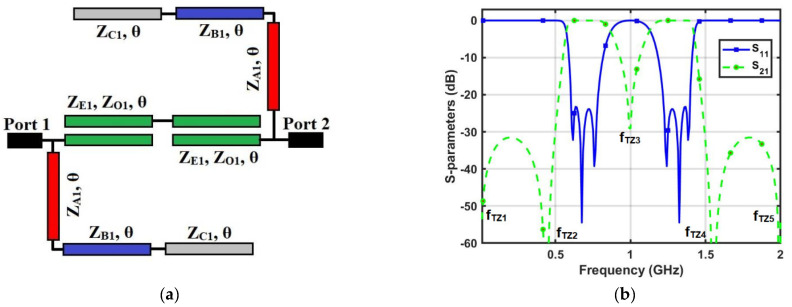
Proposed DBBPF using coupled lines and TSIOSs: (**a**) transmission line model and (**b**) S-parameters.

**Figure 2:**
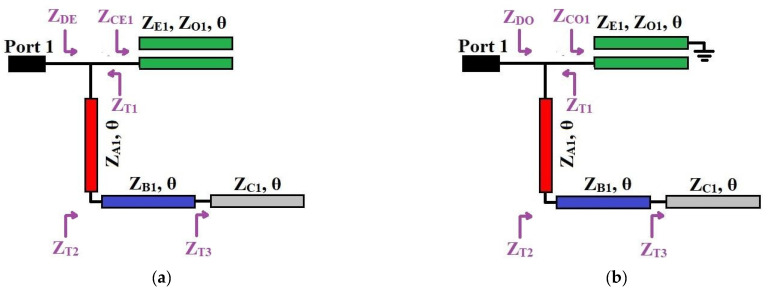
Proposed DBBPF using coupled lines and TSIOSs: (**a**) even and (**b**) odd mode equivalent circuits.

Figure 1a,b illustrates the transmission line model and S-parameters of the proposed DBBPF using coupled lines and TSIOSs. The proposed DBBPF was constructed using two coupled lines (*Z_E_*_1_, *Z_O_*_1_, *θ*) that were in series and a pair of shunt TSIOS units with impedances and electrical lengths (*Z_A_*_1_, *θ*), (*Z_B_*_1_, *θ*), and (*Z_C_*_1_, *θ*). This filter configuration, when simulated using a circuit simulator, produced two passbands with three poles in each passband and five transmission zeros in the stopband. The first and fifth transmission zeros were generated by the series-coupled lines. The second, third, and fourth transmission zeros were due to the TSIOS unit. The wideband response of the series-coupled lines overlapped with the frequency response of the TSIOSs, which contained three transmission zeros and generated two passbands. The two passbands were separated by a single transmission zero with a better than 20 dB rejection. Due to the presence of a single transmission zero, the center frequency ratio was lower, which is very useful for applications that are near. Even–odd mode analysis was incorporated to verify the proposed DBBPF. Figure 2a,b shows the even and odd mode equivalent transmission line circuits. Based on the even and odd mode admittance parameters, the S-parameters of any two-port filter configuration can be written as (1) and (2).

(1) $$S_{11}=\frac{Y_{O}^{2}-Y_{DE}Y_{DO}}{\left(Y_{O}+Y_{DE}\right)(Y_{O}+Y_{DO})}$$

(2) $$S_{21}=\frac{Y_{O}(Y_{DE}-Y_{DO})}{\left(Y_{O}+Y_{DE}\right)(Y_{O}+Y_{DO})}$$

where

(3) $$Y_{DE}=\frac{1}{Z_{DE}}=\frac{1}{Z_{T1}}+\frac{1}{Z_{CE1}}$$

(4) $$Y_{DO}=\frac{1}{Z_{DO}}=\frac{1}{Z_{T1}}+\frac{1}{Z_{CO1}}$$

(5) $$Z_{T1}=Z_{A1}\left[\frac{Z_{T2}+jZ_{A1}\tan\theta }{Z_{A1}+jZ_{T2}\tan\theta }\right]$$

(6) $$Z_{T2}=Z_{B1}\left[\frac{Z_{T3}+jZ_{B1}\tan\theta }{Z_{B1}+jZ_{T3}\tan\theta }\right]$$

(7) $$Z_{T3}=-jZ_{C1}\cot\theta$$

(8) $$Z_{CE1}=-j\left(\frac{Z_{E1}+Z_{O1}}{2}\right)\cot\theta$$

(9) $$Z_{CO1}=j\left[\frac{{\left(\frac{Z_{E1}-Z_{O1}}{2}\right)}^{2}\csc^{2}\theta -{\left(\frac{Z_{E1}+Z_{O1}}{2}\right)}^{2}\cot^{2}\theta }{\left(\frac{Z_{E1}+Z_{O1}}{2}\right)\cot\theta }\right]$$

The best way to validate the proposed filter configuration is to verify the transmission zero frequencies by setting *S*_21_ to zero, which yields *Y_DE_* = *Y_DO_*. From [13], it is well understood that the transmission zeros do not depend on series-coupled lines—they rely on shunt-connected transmission elements. Therefore, by equating the input impedance of the TSIOS unit to zero, we get the second and fourth transmission zero frequencies as:

(10) $$f_{TZ2}=\frac{f_{0}}{\theta_{0}}\tan^{-1}\sqrt{\frac{Z_{B1}Z_{C1}}{Z_{A1}\left(Z_{B1}+Z_{C1}\right)+Z_{B1}{}^{2}}}$$

(11) $$f_{TZ4}=2f_{0}-f_{TZ2}$$

**Figure 3:**
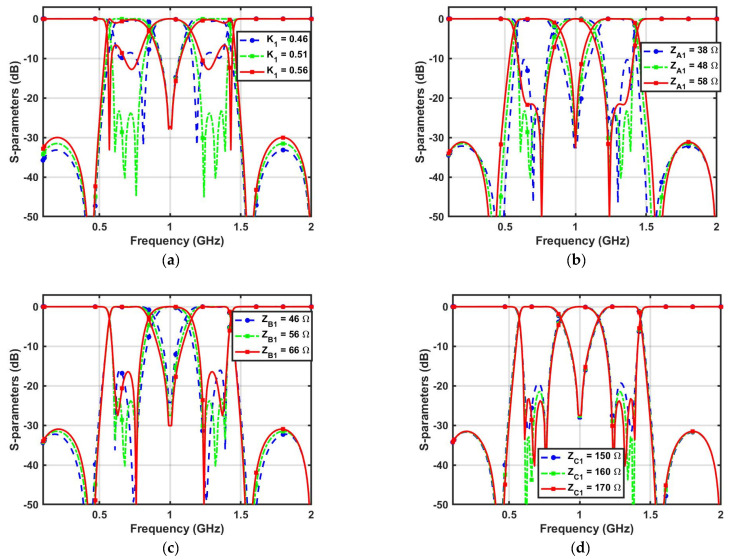
Proposed DBBPF S-parameter variations with changes in the (**a**) Coupling coefficient (*K*_1_), (**b**) *Z_A_*_1_, (**c**) *Z_B_*_1_, and (**d**) *Z_C_*_1_.

The transmission zeros at *f_TZ_*_1_ and *f_TZ_*_5_ are the inherent transmission zeros of the series-coupled lines [13]. When the electrical length tends to 90°, the denominator of (2) with higher order terms tends to infinity, giving rise to a transmission zero at *f*_0_. Considering *Z_E_*_1_ = 194 Ω, *Z_O_*_1_ = 62 Ω, *Z_A_*_1_ = 48 Ω, *Z_B_*_1_ = 56 Ω, *Z_C_*_1_ = 170 Ω, and *f*_0_ = 1 GHz, the calculated frequencies were 0, 0.44, 1, 1.56, and 2 GHz. The theoretically obtained transmission zero frequencies matched well with the circuit simulated frequencies, which validates the proposed filter configuration. The variations in the S-parameters of the proposed DBBPF using coupled lines and TSIOSs with respect to changes in the coupling coefficient (*K*_1_ = (*Z_E_*_1_ − *Z_O_*_1_)/(*Z_E_*_1_ + *Z_O_*_1_)) and impedance values (*Z_A_*_1_, *Z_B_*_1_, *Z_C_*_1_) are shown in Figure 3a–d. With increases in the K_1_ value, the stopband insertion loss decreases, and there is an effect on the passband return loss—as shown in Figure 3a. In Figure 3b, there is an improvement in the return loss of the passband when *Z_A_*_1_ increases and the second and fourth transmission zeros move away from *f*_0_. Another observation is that the inter-stopband bandwidth decreases with a good rejection level as *Z_A_*_1_ increases. When *Z_B_*_1_ increases, the inter-stopband between the passbands increases—as shown in Figure 3c. A much better passband response is obtained with increases in the impedance *Z_C_*_1_, and there is no effect on the transmission zero positions—as depicted in Figure 3d.

(b) *WBBPF using coupled lines and TSISSs*

**Figure 4:**
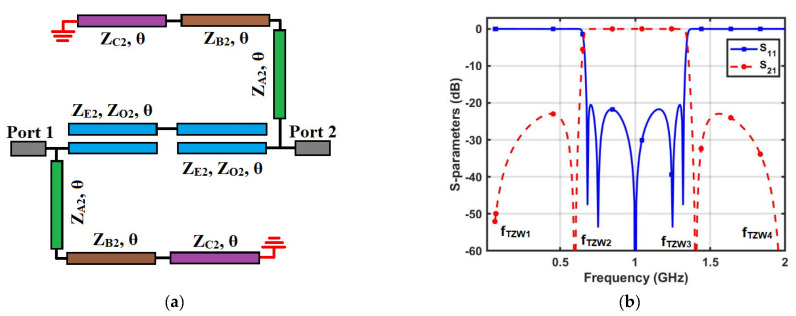
Proposed WBBPF using coupled lines and TSISSs: (**a**) transmission line model and (**b**) S-parameters.

**Figure 5:**
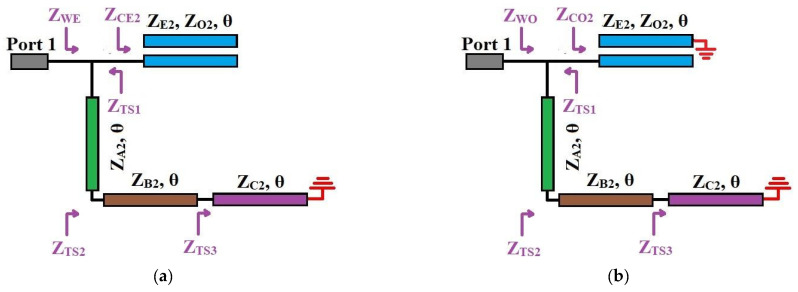
Proposed WBBPF using coupled lines and TSISSs: (**a**) even and (**b**) odd mode equivalent transmission line circuits.

A WBBPF with a high selectivity factor was proposed using series-coupled lines (*Z_E_*_2_, *Z_O_*_2_, *θ*) and a pair of shunt TSISS units with impedances and electrical lengths (*Z_A_*_2_, *θ*), (*Z_B_*_2_, *θ*), and (*Z_C_*_2_, *θ*). The transmission line model of the proposed WBBPF and its S-parameter responses are illustrated in Figure 4a,b, respectively. The frequency response contains a single wide passband with five poles and four transmission zeros in the stopband. The insertion loss in the stopband and the return loss in the passband are better than 20 dB. The circuit-simulated magnitude response showed that the proposed WBBPF using coupled lines and TSISSs had an excellent selectivity factor. Since the proposed WBBPF using coupled lines and a TSISS topology was symmetric, the analysis incorporated for the DBBPF was used here to validate the proposed WBBPF. Figure 5a,b depicts the equivalent even and odd mode units of the proposed WBBPF using coupled lines and TSISSs.

(12) $$Y_{WE}=\frac{1}{Z_{WE}}=\frac{1}{Z_{TS1}}+\frac{1}{Z_{CE2}}$$

(13) $$Y_{WO}=\frac{1}{Z_{WO}}=\frac{1}{Z_{TS1}}+\frac{1}{Z_{CO2}}$$

(14) $$Z_{TS1}=Z_{A2}\left[\frac{Z_{TS2}+jZ_{A2}\tan\theta }{Z_{A2}+jZ_{TS2}\tan\theta }\right]$$

(15) $$Z_{TS2}=Z_{B2}\left[\frac{Z_{TS3}+jZ_{B2}\tan\theta }{Z_{B2}+jZ_{TS3}\tan\theta }\right]$$

(16) $$Z_{TS3}=jZ_{C2}\tan\theta$$

(17) $$Z_{CE2}=-j\left(\frac{Z_{E2}+Z_{O2}}{2}\right)\cot\theta$$

(18) $$Z_{CO2}=j\left[\frac{{\left(\frac{Z_{E2}-Z_{O2}}{2}\right)}^{2}\csc^{2}\theta -{\left(\frac{Z_{E2}+Z_{O2}}{2}\right)}^{2}\cot^{2}\theta }{\left(\frac{Z_{E2}+Z_{O2}}{2}\right)\cot\theta }\right]$$

The first and fourth transmission zeros are the inherent transmission zeros of the coupled lines. The equations for the second and third transmission zero frequencies of the proposed WBBPF can be obtained by setting the TSISS impedance to zero to obtain:

(19) $$f_{TZW2}=\frac{f_{0W}}{\theta_{0}}\tan^{-1}\sqrt{\frac{Z_{B2}\left(Z_{A2}+Z_{B2}+Z_{C2}\right)}{Z_{A2}Z_{C2}}}$$

(20) $$f_{TZW3}=2f_{0W}-f_{TZW2}$$

**Figure 6:**
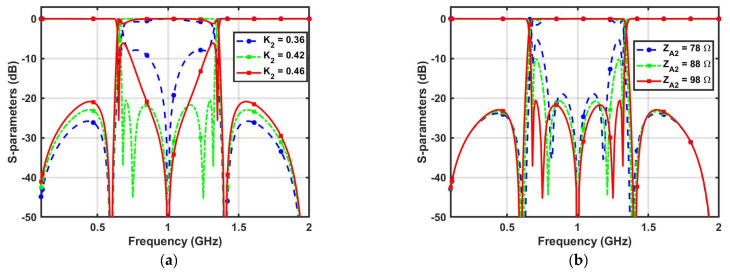

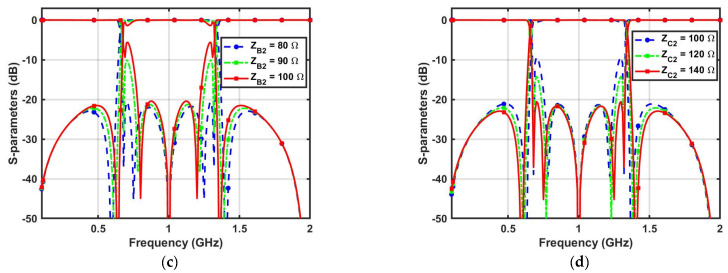
Proposed WBBPF S-parameters variations with changes in (**a**) Coupling coefficient (*K*_2_), (**b**) *Z_A_*_2_, (**c**) *Z_B_*_2_, and (**d**) *Z_C_*_2_.

Considering *Z_E_*_2_ = 196 Ω, *Z_O_*_2_ = 80 Ω, *Z_A_*_2_ = 98 Ω, *Z_B_*_2_ = 80 Ω, *Z_C_*_2_ = 140 Ω, and *f*_0*W*_ = 1 GHz, the calculated frequencies of the transmission zeros were 0, 0.60, 1.40, and 2 GHz. The determined transmission zero frequencies from (19) to (20) matched well with the circuit-simulated frequencies, validating the proposed WBBPF using coupled lines and TSISSs. Figure 6a–d shows the S-parameter variations with changes in the coupling coefficient (*K*_2_) and impedances (*Z_A_*_2_, *Z_B_*_2_, *Z_C_*_2_). With increases in the *K*_2_ value, the stopband insertion loss decreased, and there was an effect on the passband return loss—as shown in Figure 6a. The return loss in the passband increased as the value of the impedance *Z_A_*_2_ increased, and there is an insignificant effect on the transmission zero frequencies—as shown in Figure 6b. The return loss in the passband decreased as the impedance value *Z_B_*_2_ increased, and the second and third transmission zeros shifted towards the *f*_0_*—*as shown in Figure 6c. The return loss in the passband and the insertion loss in the stopband increased as the value of the impedance *Z_C_*_2_ increased. The second and third transmission zeros slightly shifted away from the *f*_0_, as shown in Figure 6d.

## 3. Results and Discussion

**Figure 7:**
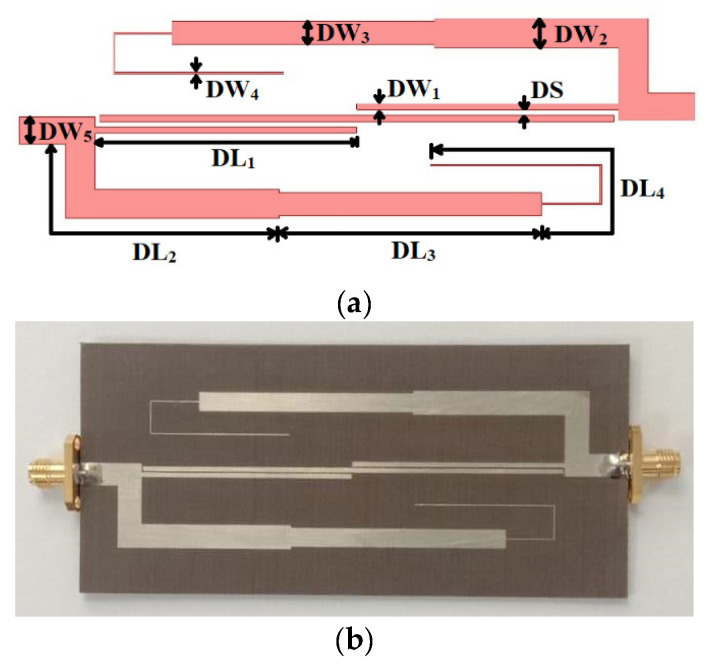
Proposed DBBPF using coupled lines and TSIOSs: (**a**) Filter layout and (**b**) manufactured prototype.

**Figure 8:**
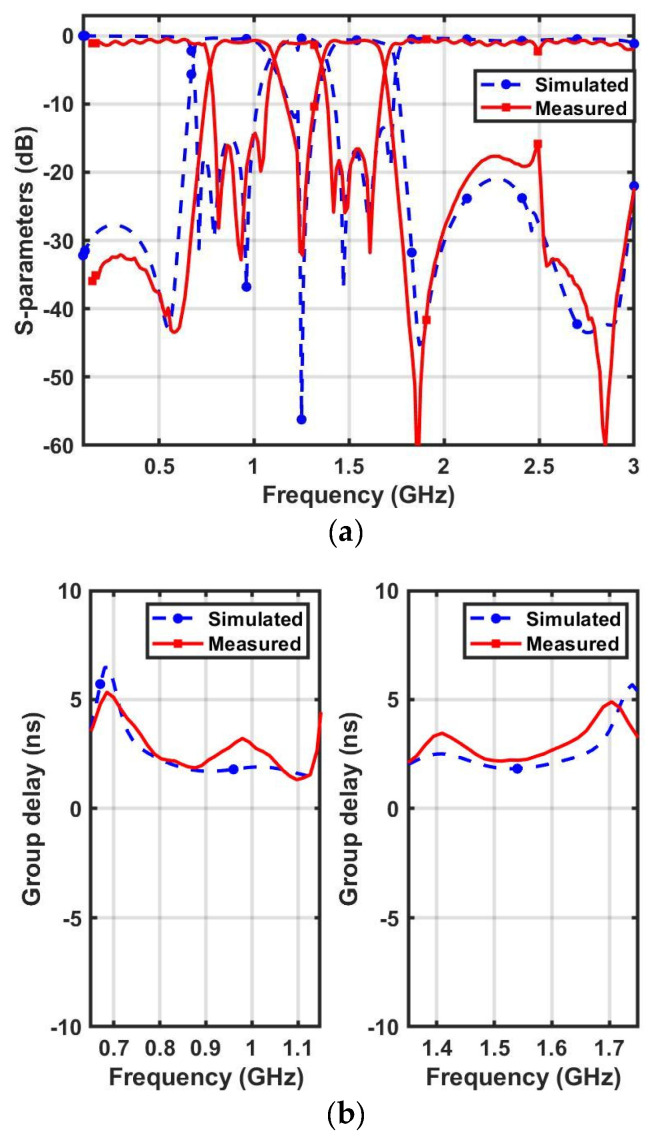
Proposed DBBPF using coupled lines and TSIOSs’ measured and simulated responses: (**a**) *S*_11_, *S*_21_, and (**b**) group delay.

The proposed dual-band and wideband bandpass filters were fabricated using a RT/duroid 5870 Rogers substrate (with a dielectric constant $\varepsilon_{r}$ of 2.33, loss tangent of 0.0012, and thickness of 1.6 mm). The design procedure reported in [13] was adopted to determine the circuit parameters. The final values of the circuit parameters for DBBPF were *Z_E_*_1_ = 194 Ω, *Z_O_*_1_ = 62 Ω, *Z_A_*_1_ = 48 Ω, *Z_B_*_1_ = 56 Ω, *Z_C_*_1_ = 170 Ω, and *f*_0_ = 1.2 GHz. Similarly, for WBBPF, the values were *Z_E_*_2_ = 196 Ω, *Z_O_*_2_ = 80 Ω, *Z_A_*_2_ = 98 Ω, *Z_B_*_2_ = 80 Ω, *Z_C_*_2_ = 140 Ω, and *f*_0*W*_ = 1.57 GHz. The proposed filters were designed and simulated in Ansys HFSS. The layout of the proposed DBBPF using coupled lines and TSISSs is shown in Figure 7a, and a photograph of the fabricated prototype is shown in Figure 7b. The dimensions were: DL_1_ = 44, DL_2_ = 49.38, DL_3_ = 44.73, DL_4_ = 45.24, DW_1_ = 1, DW_2_ = 5, DW_3_ = 3.94, DW_4_ = 0.24, DW_5_ = 4.7, and DS = 0.25 (all in mm). The filter size was 0.13 λg × 0.41 λg. The manufactured DBBPF prototype was tested using a vector network analyser, and Figure 8a,b shows the obtained results of the measured and full wave-simulated S-parameters and the group delay. The first passband-measured 3 dB bandwidth was from 0.75 to 1.1 GHz, and the center frequency was 0.92 GHz. The insertion loss and return loss at 0.92 GHz were 1.01 dB and 14 dB, respectively. The lower and upper roll-off rates for the first passband were 425 and 242 dB/GHz, respectively. The second passband-measured 3 dB bandwidth was from 1.35 to 1.69 GHz, and the center frequency was 1.52 GHz. The insertion loss and return loss at 1.52 GHz were 0.96 dB and 16 dB, respectively. The lower and upper roll-off rates for the second passband were 340 and 283 dB/GHz, respectively. The measured passband 3 dB FBWs of the proposed DBBPF were 38.04% and 22.36%. The center frequency ratio was 1.65, indicating that the two passbands were close, with a rejection better than 20 dB between them. The first and second passband group delays varied from 1.33 to 3.39 ns and 2.11 to 3.56 ns, respectively. Table 1 shows a comparison of the simulated and experimented results for the DBBPF using coupled lines and TSIOSs. The simulated and tested results were in good congruence with each other. The performance of the proposed dual-band bandpass filter was compared with published filters in Table 2 in terms of its center frequency ratio (CFR), 3 dB FBW, transmission zeros (TZs), and size. The proposed filter had a wide fractional bandwidth with a lower CFR and a comparable size when compared to most other works.

**Figure 9:**
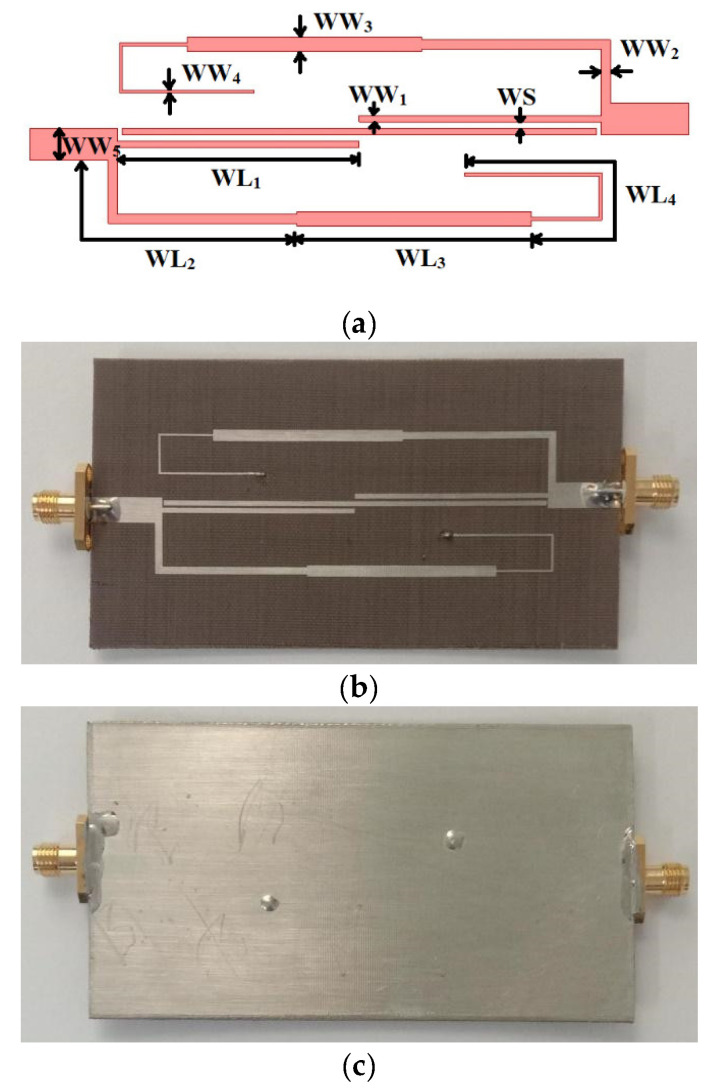
Proposed WBBPF using coupled lines and TSISSs: (**a**) Filter layout, (**b**) top, and (**c**) bottom view of the manufacured prototype.

**Figure 10:**
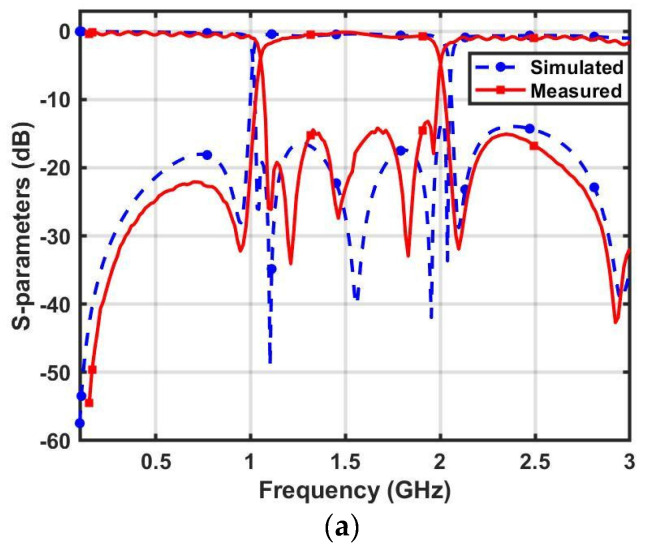

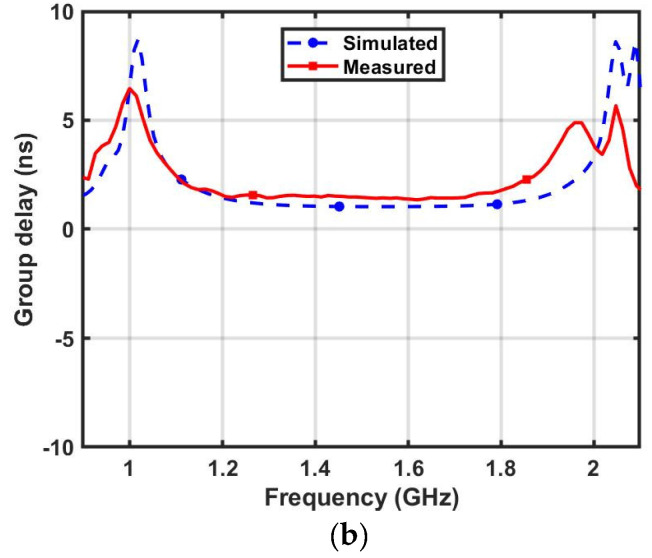
Proposed WBBPF using coupled lines and TSISSs’ measured and simulated responses (**a**) *S*_11_, *S*_21,_ and (**b**) group delay.

Figure 9a illustrates the layout of the WBBPF using coupled lines and TSISSs and Figure 9b,c shows the top and bottom views of the manufactured prototype. The dimensions were: WL_1_ = 35.4, WL_2_ = 37.37, WL_3_ = 34.83, WL_4_ = 37.41, WW_1_ = 0.94, WW_2_ = 1.37, WW_3_ = 2.1, WW_4_ = 0.41, WW_5_ = 4.7, and WS = 0.42 (all in mm). The filter size was 0.18 λg × 0.52 λg. The manufactured WBBPF prototype was tested and the obtained results were compared with full wave-simulated S-parameters and group delay in Figure 10a,b, respectively. The measured 3 dB bandwidth ranged from 1.04 to 1.99 GHz, and the center frequency was *f_C_* = 1.51 GHz. The measured 3 dB FBW was 62.91%. The insertion loss and the return loss at *f_C_* were 1.01 dB and 15 dB, respectively. The measured selectivity factor was 0.90, which indicates that the filter had a good selectivity. The measured lower and upper roll-off rates were 425 dB/GHz and 340 dB/GHz, respectively. The rejection in the lower stopband was better than 20 dB, from 0 to 1 GHz, and in the upper stopband it was better than 15 dB, from 2.04 to 3 GHz. The group delay was almost flat and ranged from 1.42 to 4.8 ns in the passband. The simulated and experimented results of the proposed WBBPF using coupled lines and TSISSs are compared in Table 3, and there is a good match between them. Table 4 shows a comparison of the proposed WBBPF using coupled lines and TSISSs with other recently reported wideband filters in terms of their 3 dB FBW, number of TZs and TPs, selectivity factor, and size. The proposed filter had a good selectivity factor, comparable bandwidth, and size when compared to most other reported works.

## 4. Conclusions

A third-order dual-band bandpass filter and a fifth-order wideband bandpass filter have been demonstrated in this paper. The design consisted of series-coupled lines and shunt tri-stepped impedance open stubs that provide a dual-passband response that is closely located. A single wide passband response with a high selectivity was obtained by replacing the shunt tri-stepped impedance open stubs with tri-stepped impedance short-circuited stubs. Both the filters were implemented on Rogers RT/duroid 5870 substrates and experimented with. The manufactured DBBPF and WBBPF prototypes occupied a circuit area of 0.13 λg × 0.41 λg and 0.18 λg × 0.52 λg, respectively. Both filters covered applications such as GSM, GPS, etc. The proposed filters based on series-coupled lines and shunt tri-stepped impedance stubs could be good candidates for modern wireless communication systems.

## Figures and Tables

**Table 1:** DBBPF using coupled lines and TSIOSs’ simulated and experimented results.

Band	Parameter	Simulated	Measured
First Passband	3 dB bandwidth (GHz)	0.68–1.08	0.75–1.1
	Center frequency (*f*_1_) GHz	0.88	0.92
	IL (dB) @ *f*_1_	0.51	1.01
	RL (dB) @ *f*_1_	15.08	14
	Roll-off rate (dB/GHz)	850/170	425/242
Second Passband	3 dB bandwidth (GHz)	1.38–1.75	1.35–1.69
	Center frequency (*f*_2_) GHz	1.56	1.52
	IL (dB) @ *f*_2_	0.64	0.96
	RL (dB) @ *f*_2_	18.16	16
	Roll-off rate (dB/GHz)	212/566	340/283

**Table 2:** DBBPF using coupled lines and TSIOSs’ performance comparison with reported DBBPFs.

Ref.	*f*_1_/*f*_2_ (GHz)	CFR	IL1/IL2 (dB)	3 dB FBW (%)	TZs	Size (λg × λg)
[1]	2.45/5.2	2.12	1.3/2.8	7/4	3	0.27 × 0.27
[4]	1.8/5.8	3.22	1.2/2.0	12.2/4.4	4	0.37 × 0.28
[6]	3.5/5.25	1.5	1.87/2.33	6.5/4.3	2	0.45 × 0.32
[7]	3.73/4.29	1.15	-	3.2/5.7	7	0.32 × 0.28
[8]	2.4/5.8	2.41	1.35/1.97	4.63/3.6	3	0.39 × 0.25
[9]	1.8/2.4	1.33	0.07/0.08	7.91/3.89	3	0.46 × 0.34
[10]	2.65/4.84	1.82	0.99/1.39	6.04/8.7	5	0.44 × 0.3
[11]	3.45/5.27	1.52	1.76/2.56	10.7/4	5	0.23 × 0.31
[12]	3.4/5.4	1.58	1.2/1.4	5.8/11.8	4	0.24 × 0.25
[13]	1.17/2.85	2.43	0.95/1.87	13.67/3.5	7	0.29 × 0.17
[14]	1.33/2.82	2.12	1.16/2.46	7.5/2.83	7	0.29 × 0.3
[15]	2.33/4.36	1.87	2/2.2	6/5.7	5	0.28 × 0.23
[16]	1.25/1.92	1.53	0.77/0.5	7.6/8.4	4	0.31 × 0.46
**This work**	**0.92/1.52**	**1.65**	**1.01/0.96**	**38.04/22.36**	**5**	**0.13 × 0.41**

**Table 3:** WBBPF using coupled lines and TSISSs’ simulated and experimented results.

Parameter	Simulated	Measured
3 dB BW range (GHz)	1.01–2.07	1.04–1.99
Center frequency (*f_C_*) GHz	1.54	1.51
IL (dB) @ *f_C_*	0.38	1.01
RL (dB) @ *f_C_*	30.41	15
Selectivity factor	0.96	0.90

**Table 4:** WBBPF using coupled lines and TSISSs’ performance comparison with reported WBBPFs.

Ref.	*f_C_* (GHz)	3 dB FBW (%)	TZs/TPs	IL (dB)	Selectivity Factor	Size (λg × λg)
[20]	3.2	20.6	6/6	2.2	0.79	1.06 × 0.61
[21]	13	48	2/6	1.1	0.88	1.97 × 0.78
[22]	2.2	10	2/5	0.9	0.70	0.26 × 0.05
[23]	3.24	58.3	2/5	0.6	0.83	0.34 × 0.34
[25]	2.5	50	2/5	0.5	0.62	--
[27]	2.1	19	8/3	1.8	0.77	0.39 × 0.28
[28]	2.7	66.6	3/3	0.8	0.77	0.20 × 0.20
[29]	6.95	97	2/3	0.42	0.69	0.128 × 0.378
[30]	3.69	73.17	4/5	1.07	0.81	0.74 × 0.94
[34]	1.25	62.4	4/3	0.9	0.69	0.23 × 0.13
**This work**	**1.51**	**62.91**	**4/5**	**1.01**	**0.90**	**0.18 × 0.52**

## References

[1] Luo X., Cheng X., Han J., Zhang L., Chen F., Guo Y., Xia X., Deng X. (2019). Compact dual-and bandpass filter using defected SRR and irregular SIR. Electron. Lett..

[2] Troudi Z., Macháč J., Osman L. (2020). Compact dual-band bandpass filter using a modified hexagonal split ring resonator. Microw. Opt. Technol. Lett..

[3] Roshani S., Yahya S.I., Mezaal Y.S., Chaudhary M.A., Al-Hilali A.A., Mojirleilani A., Roshani S. (2023). Design of a Compact Quad-Channel Microstrip Diplexer for L and S Band Applications. Micromachines.

[4] Wu X., Wan F., Ge J. (2016). Stub-loaded theory and its application to balanced dual-band bandpass filter design. IEEE Microw. Wirel. Compon. Lett..

[5] Wang Y.X., Chen Y.L., Zhou W.H., Yang W.C., Zen J. (2021). Dual-band bandpass filter design using stub-loaded hairpin resonator and meandering uniform impedance resonator. Prog. Electromagn. Res. Lett..

[6] Xie Y., Chen F.C., Li Z. (2017). Design of dual-band bandpass filter with high isolation and wide stopband. IEEE Access.

[7] Xiong Y., Wang L.T., Zhang W., Zhang F., Pang D., He M., Zhao X., Ji L. (2017). Design of dual band bandpass filter with closely spaced passbands and multiple transmission zeros. Prog. Electromagn. Res. Lett..

[8] Zhang Z.-C., Chu Q.-X., Chen F.-C. (2015). Compact dual-band bandpass filters using open-/short-circuited stub-loaded λ/4 resonators. IEEE Microw. Wirel. Compon. Lett..

[9] Song F., Wei B., Zhu L., Cao B., Lu X. (2015). Dual-band high temperature superconducting bandpass filter using quint-mode stub-loaded resonators. IEEE Trans. Appl. Supercond..

[10] Sun M., Chen Z., Zuo T., Zuo Z., Zhang A. (2021). A high selectivity dual-band bandpass filter using quadruple-mode multi-stub loaded ring resonator (SLRR). Int. J. RF Microw. Comput.-Aided Eng..

[11] Mo Y., Fan Y., Tao P., Song K. (2013). Miniaturised dual-band bandpass filter using modified SIR. Electron. Lett..

[12] Moattari A.M., Bijari A., Razavi S.M. (2021). A new compact microstrip dual bandpass filter using stepped impedance and λ/2 bended resonators. Int. J. RF Microw. Comput.-Aided Eng..

[13] Alazemi A.J. (2023). A compact diamond-shaped dual-band bandpass filter with multiple transmission zeros. AEU—Int. J. Electron. Commun..

[14] Xu J., Xu K.D., Zhang M., Chen Q. (2021). Dual-band bandpass filter using two simple coupled microstrip rings. Eng. Rep..

[15] Li D., Wang J.A., Liu Y., Chen Z., Yang L. (2021). Yang Selectivity-enhancement technique for parallel-coupled SIR based dual-band bandpass filter. Microw. Opt. Technol. Lett..

[16] Abdel-Aziz M., Anwer S., El-Hameed A., Awamry A.A., Mohra A.S. (2021). Dual-band broadside-coupled based BPF with improved performance. AEU—Int. J. Electron. Commun..

[17] Arora A., Madan A., Bhattacharjee M., Nayak C., Kumar K.V.P., Thipparaju R.R. (2020). Implementation of a compact dual-band bandpass filter using signal interference technique on paper substrate. AEU—Int. J. Electron. Commun..

[18] Shriram S.Y., Kumar K.V.P., Karthikeyan S.S. (2018). Compact dual-wideband bandpass filter for wireless applications. AEU—Int. J. Electron. Commun..

[19] Wang H., Chu Q.X., Gong J.Q. (2009). A compact wideband microstrip filter using folded multiple-mode resonator. IEEE Microw. Wirel. Compon. Lett..

[20] Feng W., Gao X., Che W., Xue Q. (2015). Bandpass filter loaded with open stubs using dual-mode ring resonator. IEEE Microw. Wirel. Compon. Lett..

[21] Nwajana A.O., Obi E.R. (2023). Application of Compact Folded-Arms Square Open-Loop Resonator to Bandpass Filter Design. Micromachines.

[22] Cheng T., Tam K.W. (2017). A wideband bandpass filter with reconfigurable bandwidth based on cross-shaped resonator. IEEE Microw. Wirel. Compon. Lett..

[23] Zheng X., Pan Y., Jiang T. (2018). UWB Bandpass Filter with Dual Notched Bands Using T-Shaped Resonator and L-Shaped Defected Microstrip Structure. Micromachines.

[24] Yang Q., Shu M., Guo C., Li J., Zhang A. (2020). High selectivity wideband bandpass filter based on stepped impedance open stubs loaded ring resonator. AEU—Int. J. Electron. Commun..

[25] Sanchez-Soriano M.A., Quendo C. (2021). Systematic design of wideband bandpass filters based on short-circuited stubs and transmission lines. IEEE Microw. Wirel. Compon. Lett..

[26] Zhang R., Zhu L. (2013). Design of a wideband bandpass filter with composite short-and open-circuited stubs. IEEE Microw. Wirel. Compon. Lett..

[27] Da Xu K., Zhang F., Liu Y., Liu Q.H. (2018). Liu Bandpass filter using three pairs of coupled lines with multiple transmission zeros. IEEE Microw. Wirel. Compon. Lett..

[28] La D.S., Guan X., Wang M.Y., Mi R.Q. (2021). Compact wideband bandpass filter based on coupled line stub with high selectivity. AEU—Int. J. Electron. Commun..

[29] Sheikhi A., Alipour A., Mir A. (2020). Design and Fabrication of an Ultra-Wide Stopband Compact Bandpass Filter. IEEE Trans. Circuits Syst. II Express Briefs.

[30] Zhang X., Wu Y., Yu H., Wang W., Yang Y., Gao J. (2022). High selectivity wideband bandpass filters based on flexibly transferring the structure of a coupled-line. AEU—Int. J. Electron. Commun..

[31] Da Xu K., Luo Z., Liu Y., Liu Q.H. (2018). High-selectivity single-ended and balanced bandpass filters using ring resonators and coupled lines loaded with multiple stubs. AEU—Int. J. Electron. Commun..

[32] Cui L., Wang W., Zhuang Z., Li S., Wu Y., Liu Y.A. (2016). High Selectivity Wideband Bandpass Filter Based on Transversal Signal-Interaction Concepts Loaded with Open and Shorted Stubs. Prog. Electromagn. Res. Lett..

[33] Kumar K.V.P., Karthikeyan S.S. (2018). Compact, high selectivity and wideband bandpass filter with multiple transmission zeros. AEU—Int. J. Electron. Commun..

[34] Narayane V.B., Kumar G. (2022). A selective wideband bandpass filter with wide stopband using mixed lumped-distributed circuits. IEEE Trans. Circuits Syst. II Express Briefs.

